# Trends in Uropathogenic *Escherichia coli* Genotype and Antimicrobial Resistance From 2019 to 2022 in a San Francisco Public Hospital Network

**DOI:** 10.1093/ofid/ofaf579

**Published:** 2025-09-17

**Authors:** Sean Joyce, Cheyenne Belmont, Aaron Wolfe Scheffler, Kavitha Ravi, Hanna Kim, Noah Rubin-Saika, Matthew Elises, Abraham Soto, Padukudru Anand Mahesh, Henry Chambers, Eva Raphael

**Affiliations:** School of Medicine, University of California San Francisco, San Francisco, California, USA; Department of Epidemiology and Biostatistics, University of California San Francisco, San Francisco, California, USA; Department of Epidemiology and Biostatistics, University of California San Francisco, San Francisco, California, USA; Center of Excellence in Molecular Biology and Regenerative Medicine Laboratory, Department of Biochemistry, JSS Medical College, JSS Academy of Higher Education and Research, Mysuru, India; School of Public Health, Yale University, New Haven, Connecticut, USA; Chicago Medical School at Rosalind Franklin University, North Chicago, Illinois, USA; University of California, Berkeley, Berkeley, California, USA; Department of Epidemiology and Biostatistics, University of California San Francisco, San Francisco, California, USA; Department of Respiratory Medicine, JSS Medical College, JSS Academy of Higher Education and Research, Mysuru, India; Department of Medicine, University of California San Francisco, San Francisco, California, USA; Department of Epidemiology and Biostatistics, University of California San Francisco, San Francisco, California, USA; Department of Family and Community Medicine, University of California San Francisco, San Francisco, California, USA

**Keywords:** antimicrobial resistance, ST131, urinary tract infections, uropathogenic *Escherichia coli*, UTI epidemiology

## Abstract

**Background:**

Uropathogenic *Escherichia coli* (UPEC) is the predominant pathogen causing urinary tract infections and frequently exhibits antimicrobial resistance (AMR). Among urinary tract infections caused by UPEC, 4 genotypes—so-called pandemic sequence types (STs)—cause >50% of infections, with ST131 particularly prone to exhibiting AMR. To investigate the role of pandemic ST prevalence in driving AMR, we prospectively collected and genotyped UPEC isolated from patient urine samples.

**Methods:**

Over separate periods in 2019 and 2022, we collected and analyzed community-onset UPEC samples from patients with bacteriuria who received care in a public health care network (N = 997). Multiplex polymerase chain reaction was used to identify the presence of UPEC pandemic STs, including ST131, while patient characteristics and antibiotic susceptibilities of the UPEC samples were collected from electronic medical records. Differences in pandemic ST prevalence and AMR between years were assessed through χ^2^ testing. Multivariable logistic regression modeling was performed for odds of AMR.

**Results:**

Pandemic ST prevalence remained stable between collection years, while resistance to any antimicrobial and trimethoprim-sulfamethoxazole generally decreased. However, within pandemic ST131, fluoroquinolone resistance increased significantly (45.6% to 76.8%, *P* < .001) even after controlling for confounding variables (adjusted odds ratio, 6.01; 95% CI, 2.41–15.01).

**Conclusions:**

Steady ST prevalence and stable or decreased AMR prevalence masked significant increased fluoroquinolone resistance with ST131, which may represent unmeasured patient characteristics, bacterial factors, or new exposure to resistant ST131 in the community. Antimicrobial surveillance of UPEC at the ST level may be important for monitoring otherwise unnoticed AMR trends.

Urinary tract infections (UTIs) are the most common infection in individuals assigned female at birth and contribute significantly to health care utilization [1, 2]. The majority of UTIs, as well as asymptomatic bacteriuria cases, are caused by uropathogenic *Escherichia coli* (UPEC) [1, 3]. These infections increasingly require the use of broad-spectrum antibiotics, as the prevalence of organisms with antimicrobial resistance (AMR) has increased worldwide [4]. UTIs can originate from community- and health care–onset infections, each with differing epidemiologic characteristics and potential risk factors [5]. Similarly, UTI risks vary by patient age and sex—for instance, with higher risks in infant males and young adult and postmenopausal females [6–8].

*E coli* exhibits significant genetic heterogeneity and can be classified into clonal groups or sequence types (STs) based on sequences of several housekeeping genes. Some STs have become so prevalent that they are referred to as “pandemic” STs [9]. Collectively, ST69, ST73, ST95, and ST131 have been the most significant pandemic lineages in recent years, accounting for nearly half of all UPEC isolates [9]. Understanding the spread of pandemic STs is important as some, particularly ST131, are associated with AMR. Pressure from antimicrobial use has also been implicated in selecting AMR within UPEC [10]. However, growing evidence suggests that common source exposures, such as ingesting food or water contaminated with resistant UPEC or having companion animals, could contribute to the proliferation of these pandemic STs [11–15]. Nevertheless, these exposures implicate only a small portion of *E coli* lineages, although including ST131; one study, for instance, attributed 8% of extraintestinal *E coli* infections to foodborne zoonotic origin [14]. As further evidence of drivers other than antimicrobial use, ST95 exhibits low levels of resistance despite remaining highly prevalent [16, 17]. In this context, further investigation of temporal trends in AMR within UPEC and pandemic STs in differing study populations may help in surveillance efforts and in understanding the variable impact of potential drivers on AMR and STs.

In this study, we aimed to investigate if there were temporal trends in UPEC pandemic ST prevalence and subsequently if there were any changes in AMR within these STs. We measured trends in pandemic STs in community-onset *E coli* bacteriuria in a San Francisco public hospital network in 2 separate isolate collection periods: 1 in 2019 and 1 in 2022. We then assessed for changes in AMR, including those within specific pandemic STs. Identifying trends in current UPEC STs and AMR could inform AMR surveillance efforts.

## METHODS

### Study Population and Isolate Collection

*E coli* clinical urine isolates were collected from the clinical microbiology laboratory of Zuckerberg San Francisco General Hospital (ZSFG), which processes samples for inpatients at ZSFG and 15 outpatient clinics in the San Francisco Health Network. Sample collection occurred from May 2019 to August 2019 and again from June 2022 to October 2022. Each clinical isolate corresponded to a urine culture growing *E coli*, which may represent asymptomatic bacteriuria or a UTI. Samples were included if they originated from community-onset infections—defined as samples collected <48 hours after inpatient admission—from patients not hospitalized in the past 90 days and from samples not collected through a catheter.

Antimicrobial susceptibilities were determined by the ZSFG clinical microbiology laboratory via Microscan and disk diffusion according to Clinical Laboratory Standards Institute guidelines [18]. Resistance profile data were subsequently obtained from patient electronic medical records (EMRs; Supplementary Table 1). Any AMR was defined as resistance to any tested antimicrobial, and resistance to an antimicrobial class was defined as resistance to at least 1 antimicrobial in that class. Multidrug resistance was defined as resistance to at least 3 classes of antimicrobials. Patient demographic characteristics considered were age group (0–17, 18–34, 35–64, and ≥65 years), sex assigned at birth, preferred language, current housing instability, and race and ethnicity. Race and ethnicity were self-reported by patients and included given inequities in AMR UTI [19]. Comorbidities included in analyses were prior antimicrobial prescription, diabetes, obesity, nephrolithiasis, previous UTI, recent vaginal infection, urinary retention, cancer, and HIV infection/AIDS. Prior antimicrobial prescription was defined as having any antimicrobial prescription documented in the EMR in the 6 months prior to UPEC culture date. These comorbidities were assessed as they were considered possible risk factors or confounders for AMR or certain pandemic STs (eg, ST131) based on prior literature [16–24].

### Laboratory Techniques and Genotyping

Single colonies of clinical isolates were cultured overnight in 2 mL of tryptic soy broth. DNA extraction from overnight cultures was performed with the Qiagen DNeasy Blood and Tissue Kit according to the accompanying protocol for bacterial DNA extraction. To ensure that samples were correctly identified as *E coli*, isolates were tested with indole, and indole-negative isolates were excluded from analysis.

A previously established and validated multiplex polymerase chain reaction method was used to assign the clinical isolates to 1 of 4 STs [25]. Briefly, this method uses 4 primer sets to amplify unique sequences found in housekeeping genes of ST69, ST73, ST95, or ST131. Gel electrophoresis was performed to visualize the presence of bands of distinct sizes corresponding to ST69, ST73, ST95, or ST131, with no band suggesting an ST other than these 4.

### Statistical Analysis

We assessed differences in ST distribution and AMR between the 2019 and 2022 collection cohorts. We focused our analysis on clinically relevant antimicrobials: fluoroquinolones, trimethoprim-sulfamethoxazole, nitrofurantoin, cephalexin, any AMR, multidrug resistance, and extended-spectrum β-lactamase (ESBL) production. Changes in ST distribution between the 2019 and 2022 collection periods were assessed with χ^2^ or Fisher exact testing as appropriate. We then determined changes in AMR between 2019 and 2022 among all isolates and within each ST through χ^2^ testing. To further assess AMR changes in the context of different patient characteristics, adjusted odds ratios (ORs) of AMR within and across STs were obtained through multivariable logistic regressions. We first conducted univariate regression analyses with collected covariates for odds of any AMR (Supplementary Table 2). Prior antibiotics (OR, 1.55; *P* = .002) and previous UTI (OR, 1.62; *P* = .001) were both significantly associated with increased odds of any resistance in univariate analysis and changed significantly in prevalence between 2019 and 2022; therefore, each was selected for inclusion in the multivariable logistic regression model. Previous vaginal infection and housing instability were also included as covariates. Although they did not achieve significance in univariate analyses, they were the only 2 covariates to increase significantly in prevalence between 2019 and 2022 and could thus theoretically contribute to any observed increases in AMR. We employed Firth's bias reduction method in our multivariable logistic regression models given limited separation in our data for some antimicrobial and ST classes [26]. Limited separation occurs when a predictor nearly perfectly predicts an outcome—for instance, in our data when AMR prevalence approaches 100%. In these instances, Firth's method helps in reducing bias in estimates.

All reported confidence intervals are 95% CIs. Confidence intervals for AMR prevalence are 95% Wilson score intervals for proportions. ORs are obtained from univariate or multivariable logistic regression models, where appropriate.

### Patient Consent Statement

This study was conducted with patient consent and approved by the institutional review boards at UCSF and ZSFG (19-27233).

## RESULTS

### Patient Characteristics With Bacteriuria Caused by UPEC

We collected and genotyped 551 *E coli* urine isolates in 2019 and 446 isolates in 2022 from patients with community-onset bacteriuria. We first described patient demographic characteristics and comorbidities in these cohorts and assessed differences through χ^2^ testing (Table 1). Patients from the 2022 cohort as compared with 2019 had higher proportions of current housing instability and previous vaginal infection and lower proportions of obesity, urinary retention, previous UTI, diabetes, prior antibiotics, and HIV infection/AIDS.

**Table 1.** Characteristics of Patients Who Experienced Community-Onset Bacteriuria Caused by *Escherichia coli*: 2019 and 2022

	Patients, No. (%)		
	2019 (n = 551)	2022 (n = 446)	*P* Value
Sex assigned at birth			.304
Female	462 (83.8)	381 (85.4)	
Male	89 (16.2)	61 (13.7)	
Not provided	0 (0)	<5 (<X%)	
Age category, y			.548
0–17	25 (4.5)	20 (4.5)	
18–34	126 (22.9)	119 (26.7)	
35–64	251 (45.6)	194 (43.5)	
≥65	149 (27.0)	113 (25.3)	
Language			.076
English	314 (57.0)	221 (49.6)	
Chinese dialect	29 (5.3)	35 (7.8)	
Other	24 (4.4)	22 (4.9)	
Spanish	179 (32.5)	166 (37.2)	
No data	5 (0.9)	<5 (<X%)	
Race			.728
White	97 (17.6)	82 (18.4)	
American Indian or Alaska Native	9 (1.6)	<5 (<X%)	
Asian	97 (17.6)	74 (16.6)	
Black or African American	65 (11.8)	46 (10.3)	
Native Hawaiian or other Pacific Islander	7 (1.3)	<5 (<X%)	
Other	259 (47.0)	213 (47.8)	
Not provided	17 (3.1)	24 (5.4)	
Ethnicity			.625
Latine	262 (47.6)	223 (50.0)	
Not Latine	274 (49.7)	219 (49.1)	
Not provided	15 (2.7)	4 (0.9)	
Current housing instability	13 (2.4)	57 (12.8)	<.001
Obesity	60 (10.9)	26 (5.8)	.005
Nephrolithiasis	23 (4.2)	12 (2.7)	.206
Urinary retention	39 (7.1)	13 (2.9)	.003
Previous urinary tract infection	214 (38.8)	107 (24.0)	<.001
Previous vaginal infection	11 (2.0)	23 (5.2)	.011
Prior antibiotics	245 (44.5)	164 (36.8)	.014
Diabetes	146 (26.5)	84 (18.8)	.004
Cancer	46 (8.3)	24 (5.4)	.068
HIV infection/AIDS	22 (4.0)	0 (0)	<.001

Patient characteristics were obtained from electronic medical record data. *P* values are based on χ^2^ results for community-onset bacteriuria between 2019 and 2022. Groups with <5 individuals are masked without percentages to protect patient anonymity.

### Pandemic ST Prevalence and Time Trends

The 4 pandemic STs that we assessed accounted for just under half of all STs across the community-onset cohorts: 46.8% in 2019 infections and 47.5% in 2022 infections (Table 2). We also compared ST distribution among age categories, as well as by sex assigned at birth. In χ^2^ testing, male sex assigned at birth as compared with female or undisclosed did not have a significantly different ST distribution (*P* = .090). Age groups, however, showed significantly variable distributions in pandemic ST, with ages 0 to 17 years (*P* = .017), 35 to 64 (*P* = .007), and ≥65 (*P* < .001) significantly different in pairwise χ^2^ when tested against all other ages. Within these age categories, ST131 was less prevalent in ages 0 to 17 (*P* = .023) and more prevalent in ages 35 to 64 (*P* = .003). Between collection years, isolates between 2019 and 2022 did not show significant differences in ST distribution in χ^2^ testing (*P* = .860). As a secondary analysis, we assessed differences in ST distribution between collection years within each age category in pairwise χ^2^. Results were nonsignificant in each age category and are shown in Supplementary Table 3.

**Table 2.** *Escherichia coli* Pandemic and All Other Sequence Type Prevalence in Community-Acquired Bacteriuria Isolates

	Collection Year		Sex Assigned at Birth		Age Category, y			
ST	2019 (n = 551)	2022 (n = 446)	Female or Undisclosed (n = 847)	Male (n = 150)	0–17 (n = 45)	18–34 (n = 245)	35–64 (n = 445)	≥65 (n = 262)
ST69	54 (9.8)	51 (11.4)	97 (11.5)	8 (5.3)	9 (20.0)	31 (12.7)	54 (12.1)	11 (4.2)
ST73	54 (9.8)	45 (10.1)	79 (9.3)	20 (13.3)	8 (17.8)	26 (10.6)	47 (10.6)	18 (6.9)
ST95	71 (12.7)	60 (13.5)	113 (13.3)	18 (12.0)	4 (8.9)	39 (15.9)	50 (11.2)	38 (14.5)
ST131	79 (14.3)	56 (12.6)	110 (13.0)	25 (16.7)	1 (2.2)	26 (10.6)	76 (17.1)	32 (12.2)
Other	293 (53.2)	234 (52.5)	448 (52.9)	79 (52.7)	23 (51.1)	123 (50.2)	218 (49.0)	163 (62.2)

Data are presented as No. (%) of patients. Number of isolates and percentage of STs as determined via multiplex polymerase chain reaction, by collection year, sex assigned at birth, or age category.

Abbreviation: ST, sequence type.

Among patients with multiple isolates in a collection year (n = 169), ST distribution differed significantly (*P* = .015) due to a higher prevalence of the heterogenous “other” STs (61.5% vs 51.1%), while ST131 prevalence was similar (15.4% vs 13.2%). Of 32 such patients with ≥1 pandemic ST, half (n = 16) had differing STs.

### Changes in AMR in Community-Onset Bacteriuria

**Figure 1.**
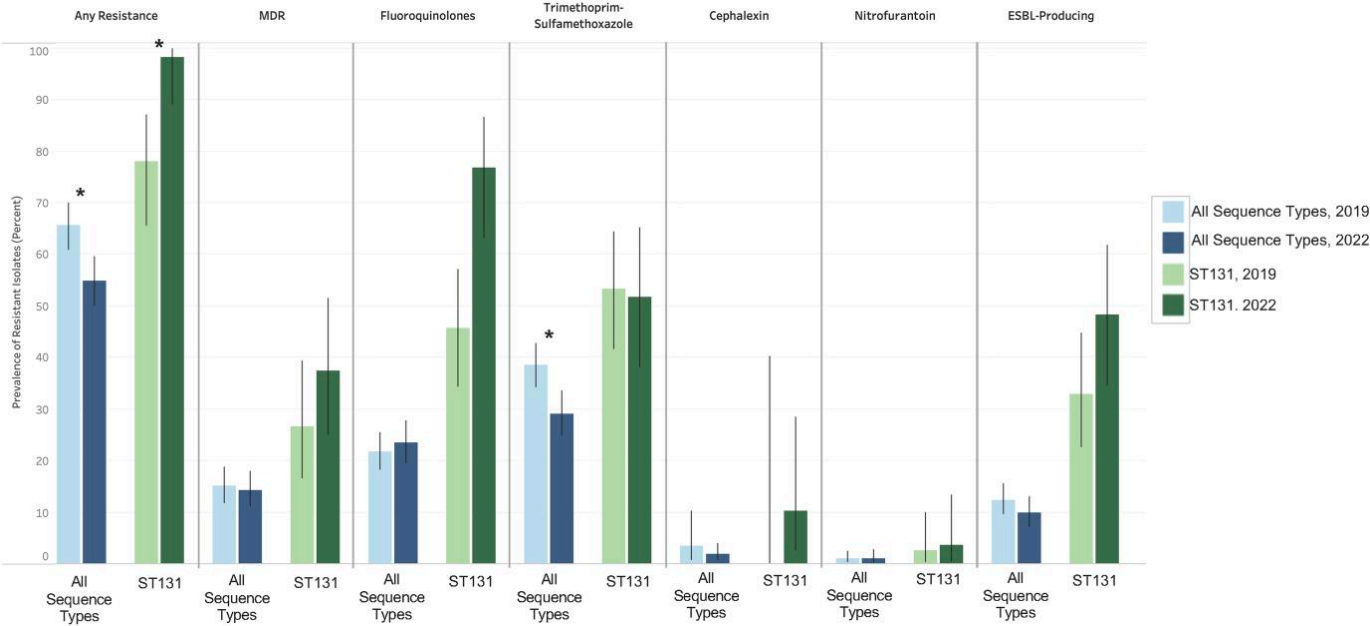
Antimicrobial-resistant isolates in community-onset bacteriuria episode: all sequence types (STs) and within ST131. AMR of uropathogenic *Escherichia coli* isolates is presented for select antimicrobial agents. Data are categorized by collection year (color) and ST (across all STs or within ST131 only). *P < .05. *P* values are from pairwise χ^2^ comparing AMR between collection years. Bars represent 95% CIs for a proportion. Abbreviations: AMR, antimicrobial resistance; ESBL, extended-spectrum β-lactamase; MDR, multidrug resistance.

We determined the prevalence of AMR within STs and assessed significant changes between collection years within community-onset bacteriuria (Supplementary Table 4). Confidence intervals denote the 95% Wilson score interval for a proportion. Among all isolates, 65.8% (95% CI, 61.0%–70.0%) from 2019 and 54.9% (95% CI, 50.2%–59.6%) from 2022 displayed any AMR, a significant decrease in χ^2^ testing (*P* < .001; Figure 1). In 2019 and 2022, 15.1% (95% CI, 11.9%–18.8%) and 14.3% (95% CI, 11.3%–18.0%) exhibited multidrug resistance, respectively (*P* = .766). Trimethoprim-sulfamethoxazole resistance also decreased significantly (*P* = .002) from 38.5% (95% CI, 34.4%–42.7%) in 2019 to 29.1% (95% CI, 25.0%–33.6%) in 2022. Fluoroquinolone resistance was 21.8% (95% CI, 18.4%–25.5%) in 2019 and 23.5% (95% CI, 19.7%–27.8%) in 2022, not significantly different (*P* = .508). In contrast to these decreases in resistance across all isolates, ST131 isolates displayed increased resistance to fluoroquinolones and any antimicrobial. Within ST131 isolates, fluoroquinolone resistance rose from 45.6% (95% CI, 34.5%–57.1%) to 76.8% (95% CI, 63.3%–86.6%; *P* < .001). ST131 exhibited a very high prevalence to any antimicrobial, increasing from 78.1% in 2019 (95% CI, 65.7%–87.1%) to 98.2% in 2022 (95% CI, 89.2%–99.9%; *P* < .001).

No other ST displayed an increase in AMR, although significant decreases primarily within “other” STs were noted (Supplementary Table 4). Within these other STs, any AMR (68.4% in 2019 [95% CI, 61.9%–74.3%] vs 52.6% in 2022 [95% CI, 46.0%–59.1%], *P* < .001) and trimethoprim-sulfamethoxazole (38.6% in 2019 [95% CI, 33.0%–44.4%] vs 29.1% in 2022 [95% CI, 23.4%–35.4%], *P* = .022) decreased and therefore likely drove the observed decreases among all isolates in any antimicrobial and trimethoprim-sulfamethoxazole resistance.

**Figure 2.**
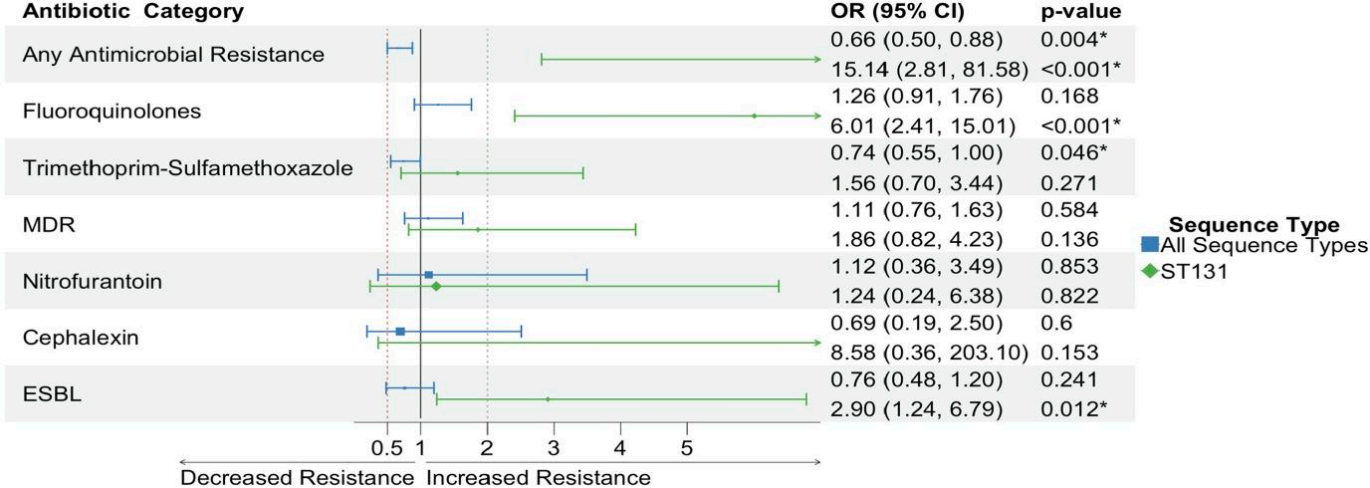
Adjusted odds ratios (ORs) of antimicrobial resistance between 2019 and 2022 across all sequence types and within ST131. Adjusted OR is for antimicrobial resistance to an antimicrobial class per collection year. Multivariable logistic regression models were adjusted for prior antibiotics, previous urinary tract infection, housing instability, and previous vaginal infection. *P < .05. Abbreviations: ESBL, extended-spectrum β-lactamase; MDR, multidrug resistance.

Significant differences in patient characteristics between 2019 and 2022 could contribute to the rise in fluoroquinolone resistance within ST131 isolates, depressed AMR among all isolates, or both (Table 1). In multivariable logistic regression models adjusting for housing instability, previous vaginal infection, prior antibiotics, and previous UTI, odds of fluoroquinolone resistance remained higher within ST131 in 2022 (OR, 6.01; 95% CI, 2.41–15.01; *P* < .001; Figure 2). Among all isolates, odds of fluoroquinolone resistance was higher as well, although this did not achieve statistical significance (OR, 1.26; 95% CI, .91–1.76; *P* = .168).

Adjusted odds of any AMR in 2022 was significantly higher within ST131 isolates (OR, 15.4; 95% CI, 2.81–81.58; *P* < .001) yet significantly lower among all isolates (OR, 0.66; 95% CI, .50–.88; *P* = .004; Figure 2). While a significant difference, the high prevalence in any AMR within ST131 and the subsequent lack of separation in our data limit our ability to comment on the magnitude of this relationship, even after applying Firth's bias reduction method. Adjusted odds of trimethoprim-sulfamethoxazole resistance was additionally lower among all isolates (OR, 0.74; 95% CI, .55–1.00; *P* = .046) while nonsignificant within ST131. We also observed increased adjusted odds of ESBL production in ST131 (OR, 2.90; 95% CI, 1.24–6.79; *P* = .012), which was not seen among all isolates. Odds of multidrug resistance, nitrofurantoin, and cephalexin were not significant among all isolates or within ST131. Full adjusted ORs for ST69, ST73, and ST95 are provided in Supplementary Figure 1.

Full ORs for the covariates included in these multivariable analyses are shown in Supplementary Table 5. Of note, prior antibiotics were a risk factor for fluoroquinolone resistance among all isolates (OR, 1.72; 95% CI, 1.22–2.43) and within ST131 (OR, 3.04; 95% CI, 1.24–7.46). Increased odds of any resistance (OR, 3.81; 95% CI, 1.15–12.68) and resistance to trimethoprim-sulfamethoxazole (OR, 2.90; 95% CI, 1.31–6.44) with prior antibiotics were also seen within ST131 isolates but not overall.

## DISCUSSION

In this study, we observed a stable prevalence of pandemic STs in *E coli* isolates from community-onset bacteriuria in a public hospital and clinic network. ST distribution varied by age group, with ST131 more prevalent in ages 35 to 64 years. Any AMR and trimethoprim-sulfamethoxazole resistance decreased in community-onset isolates between 2019 and 2022 overall, largely due to decreases within the prevalent “other” ST group. However, any AMR and fluoroquinolone resistance increased significantly within ST131 isolates over the 2 collection periods. These associations held after controlling for potential confounders in patient characteristics (fluoroquinolone: OR, 6.01; 95% CI, 2.41–15.01; *P* < .001). We also observed increases in adjusted odds of ESBL production within ST131. Multidrug, nitrofurantoin, and cephalexin resistances did not differ significantly between collection years across or within any ST.

Our analysis of UPEC pandemic ST distribution provides information on temporal trends in these STs at an intermediate time frame. One study over a 17-year period of urine *E coli* isolates in a college community near our study population found changes in prevalence of ST69, ST73, ST95, and ST131 of only a few percentage points, with a final ST131 prevalence of 5.2% in 2017 [27]. Yet in other populations and periods, ST prevalence changed more rapidly, such as the initial rise of ST131 to 20% prevalence in the United States in <10 years or with significant changes in UPEC strain phenotype within just 1 year in a study in Peru [28, 29]. However, we found no significant difference in pandemic ST distribution between 2019 and 2022. This includes ST69, ST95, and ST131, which have broadly been tied to possible foodborne exposure in varied geographic contexts and accounted for 37.5% of 2022 isolates [11, 15]. In analyzing our ST trends, we considered the potential impact of patients contributing multiple isolates, which could represent colonization or continuation of an infection with 1 ST. However, these were characterized by an increased prevalence of the heterogenous “other” ST category as well as variable ST representation within individual patients, suggesting against bias from repeat infections from colonized cases.

The observed fluroquinolone resistance in our study was high when compared with other studies, at 23.5% overall and 76.8% in ST131 in 2022 [27, 30]. Rising drug resistance in UPEC, particularly fluroquinolones, has been noted worldwide [31]. While the stable or declining overall AMR prevalence in our cohort is reassuring, the increase in resistance within ST131 raises concern. Given the high initial fluoroquinolone resistance in our study, prescription of broad-spectrum cephalosporins for treatment could select for ESBL production associated with fluoroquinolone resistance [32–34]. However, as we did not assess antimicrobial prescription patterns, alternative explanations include common source exposure to resistant UPEC or changes in unidentified risk factors [11–14]. Regardless, this increase in fluoroquinolone resistance within ST131 did not reach significance among all isolates, while the increase in any AMR in ST131 was also masked by an overall decrease in any AMR among all STs. The presence of these concerning increases only within ST131 indicates a need for ongoing assessment and monitoring of specific UPEC STs.

Our study has several limitations. First, our 2022 collection followed the peak of the COVID-19 outbreak. Subsequent barriers to health care access may mean that patient comorbidities are underassessed in the 2022 collection cohort. We cannot differentiate this from overall reductions in comorbidities from 2019 to 2022 or from increased *E coli* bacteriuria incidence in healthier populations. Regardless, we controlled for potentially confounding comorbidities in our analysis. Second, we could compare time trends within only a 3-year period. A longer study period might provide additional information on long-term fluctuations and rates of change of ST prevalence. Third, our genotyping method identified only ST69, ST73, ST95, and ST131, so we were not able to assess the prevalence of emerging STs such as ST1193 [35]. While validated with high sensitivity and specificity, this method may still produce some misclassification of STs [36]. Similarly, when compared with more comprehensive genotyping methods, such as whole genome sequencing, multiplex polymerase chain reaction cannot identify single- or double-locus variants, which for instance may have a role in ST131 carbapenem resistance epidemiology [37]. We are cannot assess for sublineages within STs. Differentiation of specific ST sublineages is important for understanding UPEC epidemiology—for instance, where ST131-H30 is particularly implicated in AMR, while ST131-H22 is most associated with foodborne origin [38]. However, this method enabled us to analyze a larger sample size while still capturing important STs. Fourth, susceptibility data were obtained from the EMR, rather than directly tested for by the study team with reference methods, which could introduce errors in susceptibilities. Yet, Clinical Laboratory Standards Institute standards aim for robust ≥90% categorical agreement in antimicrobial susceptibility testing, which is routinely met for *E coli* [39–42]. The EMR data also do not capture prior antimicrobial use data from outside our health care system. Nevertheless, other studies support robust in-network retention in our study population [43–45]. Fifth, while our study assesses *E coli* found in urine, it does not distinguish between asymptomatic *E coli* bacteriuria and symptomatic UTI, which may have differences in prevalent STs [46].

Despite these limitations, our study has several strengths and significant contributions. This study assessed current AMR and ST prevalence in a diverse safety net hospital system. Results therefore represent UPEC epidemiology in an understudied population with significant UTI disparities [47]. As we additionally had robust patient-level comorbidity data, we were better able to control for changes in study population characteristics. In finding a rapid increase in fluoroquinolone AMR within ST131 alongside an overall decrease in resistance, this study importantly demonstrates that routine surveillance of AMR in bacteriuria may miss significant changes in clinically relevant strains. As assessed patient-level characteristics did not sufficiently account for increases in AMR within ST131, additional studies are needed to understand possible exposures, including antimicrobial prescription, bacterial factors, or patient behaviors and characteristics that could contribute to ST-specific AMR trends.

## CONCLUSION

In this study of UPEC isolates from bacteriuria cases in a public hospital and associated clinics in San Francisco, California, the prevalence of UPEC pandemic STs did not change significantly between 2019 and 2022 in community-onset bacteriuria isolates. Overall resistance to any antimicrobial and to trimethoprim-sulfamethoxazole decreased, primarily within STs besides the pandemic ST69, ST95, and ST73, although resistance to any antimicrobial and specifically to fluoroquinolones increased within ST131 after adjusting for patient comorbidities. The increased fluoroquinolone resistance prevalence within ST131 despite overall decreases in resistance to any antimicrobial highlights the importance of surveillance of individual STs within UPEC.

## Supplementary Material

ofaf579_Supplementary_Data

## Supplementary Data

Supplementary materials are available at *Open Forum Infectious Diseases* online. Consisting of data provided by the authors to benefit the reader, the posted materials are not copyedited and are the sole responsibility of the authors, so questions or comments should be addressed to the corresponding author.
