# Supplementary material for: Trends in Uropathogenic Escherichia coli Genotype and Antimicrobial Resistance From 2019 to 2022 in a San Francisco Public Hospital Network
Source: Open Forum Infect Dis. 2025 Sep 17;12(9):ofaf579. doi: 10.1093/ofid/ofaf579 (PMC12464484; doi:10.1093/ofid/ofaf579)
Supplement: ofaf579_Supplementary_Data [file ofaf579_supplementary_data.zip › Supplemental_Figure_1.docx]

Supplemental Figure 1: Adjusted odds ratios of antimicrobial resistance for each sequence type


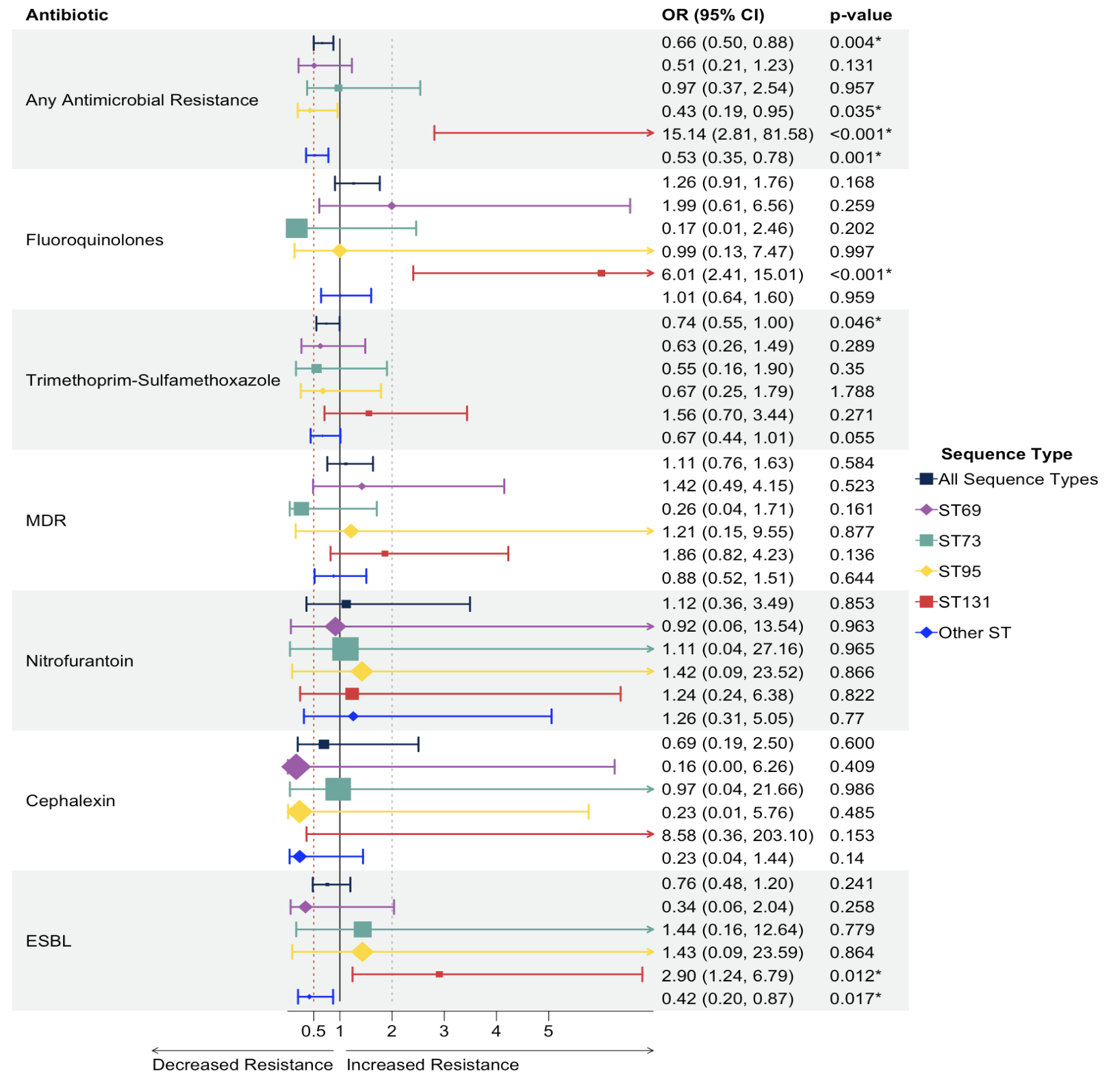


Note: Adjusted odds ratios are odds ratio of antimicrobial resistance to given antimicrobial class for collection year. Controlled for prior antibiotics, previous UTI, housing instability, and previous vaginal infection. * indicates P values <0.05.
