# Supplementary material for: Trends in Uropathogenic Escherichia coli Genotype and Antimicrobial Resistance From 2019 to 2022 in a San Francisco Public Hospital Network
Source: Open Forum Infect Dis. 2025 Sep 17;12(9):ofaf579. doi: 10.1093/ofid/ofaf579 (PMC12464484; doi:10.1093/ofid/ofaf579)
Supplement: ofaf579_Supplementary_Data [file ofaf579_supplementary_data.zip › Supplemental_Table_1.docx]

**Supplemental Table 1:** Antimicrobial agents and corresponding antimicrobial classes assessed for resistance

| Antimicrobial Class | Antimicrobial Agents |
| --- | --- |
| Aminoglycosides | Gentamicin |
|  | Streptomycin |
|  | Tobramycin |
| Carbapenems | Ertapenem |
|  | Imipenem |
|  | Meropenem |
| Cephalosporins | Cefazolin |
|  | Cephalexin |
|  | Cefuroxime |
|  | Ceftazidime |
|  | Ceftriaxone |
|  | Cefotaxime |
|  | Cefepime |
| Macrolides | Azithromycin |
|  | Erythromycin |
| Penicillins | Penicillin |
|  | Ampicillin |
|  | Nafcillin-oxacillin |
| Beta-lactamase inhibitor combinations | Amoxacillin-clavulanate |
|  | Ampicillin-sulbactam |
|  | Piperacillin-tazobactam |
|  | Ceftolozane-tazobactam |
| Quinolones | Ciprofloxacin |
|  | Levofloxacin |
|  | Moxifloxacin |
| Tetracyclines | Doxycycline |
|  | Tetracycline |
| Glycopeptides | Vancomycin |
| Oxazolidinones | Linezolid |
| Rifamycins | Rifampin |
| Sulfonamides combination | Trimethoprim-sulfamethoxazole |
| Lincosamides | Clindamycin |
| Lipopeptides | Daptomycin |
| Nitrofuran derivate | Nitrofurantoin |
| Nitro-imidazole derivatives | Metronidazole |
| Phosphonic | Fosfomycin |
| Amphenicols | Chloramphenicol |
