# Supplementary material for: Trends in Uropathogenic Escherichia coli Genotype and Antimicrobial Resistance From 2019 to 2022 in a San Francisco Public Hospital Network
Source: Open Forum Infect Dis. 2025 Sep 17;12(9):ofaf579. doi: 10.1093/ofid/ofaf579 (PMC12464484; doi:10.1093/ofid/ofaf579)
Supplement: ofaf579_Supplementary_Data [file ofaf579_supplementary_data.zip › Supplemental_Table_2.docx]

**Supplemental Table 2:** Univariate logistic regression results for odds of any antimicrobial resistance among all isolates

| **Characteristic** | **N** | **OR** | **95% CI** | **p-value** |
| --- | --- | --- | --- | --- |
| **Collection Year** | 891 |  |  | **0.001** |
| 2019 |  | — | — |  |
| 2022 |  | 0.64 | 0.49, 0.84 |  |
| **Age Category** | 891 |  |  | 0.417 |
| 0-17 |  | — | — |  |
| 18-34 |  | 1.60 | 0.82, 3.15 |  |
| 35-64 |  | 1.71 | 0.90, 3.28 |  |
| 65+ |  | 1.52 | 0.78, 2.98 |  |
| **Language** | 889 |  |  | **0.022** |
| English |  | — | — |  |
| Chinese Dialect |  | 1.04 | 0.61, 1.81 |  |
| Other |  | 0.60 | 0.32, 1.12 |  |
| Spanish |  | 1.41 | 1.05, 1.90 |  |
| **Race** | 856 |  |  | **0.034** |
| White |  | — | — |  |
| American Indian or Alaska Native |  | 6.88 | 1.27, 128 |  |
| Asian |  | 0.79 | 0.51, 1.24 |  |
| Black or African American |  | 0.83 | 0.49, 1.40 |  |
| Native Hawaiian or Other Pacific Islander |  | 1.38 | 0.35, 6.71 |  |
| Other |  | 1.22 | 0.84, 1.77 |  |
| **Ethnicity** | 881 |  |  | 0.102 |
| Not Latine |  | — | — |  |
| Latine |  | 1.25 | 0.96, 1.64 |  |
| **Sex Assigned at Birth** | 887 |  |  | 0.188 |
| Female |  | — | — |  |
| Male |  | 1.32 | 0.88, 2.00 |  |
| **Previous Vaginal Infection** | 891 | 0.73 | 0.37, 1.47 | 0.377 |
| **Prior Antibiotics** | 891 | 1.55 | 1.18, 2.05 | **0.002** |
| **Obesity** | 891 | 1.06 | 0.66, 1.72 | 0.810 |
| **Current Housing Instability** | 891 | 1.48 | 0.89, 2.54 | 0.134 |
| **Nephrolithiasis** | 891 | 4.13 | 1.73, 12.2 | **<0.001** |
| **Urinary Retention** | 891 | 1.95 | 0.94, 4.45 | 0.076 |
| **HIV Infection/AIDS** | 891 | 0.65 | 0.22, 1.93 | 0.434 |
| **Cancer** | 891 | 1.14 | 0.68, 1.94 | 0.630 |
| **Diabetes** | 891 | 1.16 | 0.84, 1.61 | 0.383 |
| **Previous UTI** | 891 | 1.62 | 1.21, 2.19 | **0.001** |
