# Supplementary material for: Trends in Uropathogenic Escherichia coli Genotype and Antimicrobial Resistance From 2019 to 2022 in a San Francisco Public Hospital Network
Source: Open Forum Infect Dis. 2025 Sep 17;12(9):ofaf579. doi: 10.1093/ofid/ofaf579 (PMC12464484; doi:10.1093/ofid/ofaf579)
Supplement: ofaf579_Supplementary_Data [file ofaf579_supplementary_data.zip › Supplemental_Table_3.docx]

Supplemental Table 3: Pandemic ST distribution in 2019 and 2022, by age category

Note: Table shows number of isolates for each sequence type in 2019 and 2022, by age category. Percentages are shown in parentheses (%). The bottom row p values are the results of pairwise Chi squared analyses comparing sequence type distribution in 2019 to 2022, for each age category.
