# Supplementary material for: Trends in Uropathogenic Escherichia coli Genotype and Antimicrobial Resistance From 2019 to 2022 in a San Francisco Public Hospital Network
Source: Open Forum Infect Dis. 2025 Sep 17;12(9):ofaf579. doi: 10.1093/ofid/ofaf579 (PMC12464484; doi:10.1093/ofid/ofaf579)
Supplement: ofaf579_Supplementary_Data [file ofaf579_supplementary_data.zip › Supplemental_Table_4.docx]

Supplemental Table 4: Antimicrobial resistance prevalence by sequence type, 2019 and 2022

|  | ST69 | | | ST73 | | | ST95 | | | ST131 | | | Other | | | All Sequence Types | | |
| --- | --- | --- | --- | --- | --- | --- | --- | --- | --- | --- | --- | --- | --- | --- | --- | --- | --- | --- |
|  | 2019 | 2022 | p-value | 2019 | 2022  (n=45) | p-value | 2019 | 2022 | p-value | 2019 | 2022 | p-value | 2019 | 2022 | p-value | 2019 | 2022 | p-value |
| Fluoroquinolones | 5  (9.3%, CI: 3.5% - 21.1%) | 9 (17.6%, CI: 8.9% - 31.4%) | 0.206 | 3 (5.6%, CI: 1.4% - 16.3%) | 0 (0.0%, CI: 0.0% - 9.8%] | 0.249 | 1 (1.4%, CI: 0.1% - 8.7%) | 1 (1.7%, CI: 0.1% - 10.1%) | 1 | **36 (45.6%, CI: 34.5% - 57.1%)** | **43 (76.8%, CI: 63.3% - 86.6%)** | **<0.001** | 75 (25.6%, CI: 20.8% - 31.1%) | 52 (22.2%, CI: 17.2% - 28.2%) | 0.368 | 120 (21.8%, CI: 18.4% - 25.5%) | 105 (23.5%, CI: 19.7% - 27.8%) | 0.508 |
| Any Resistance | 30 (71.4%, CI: 55.2% - 83.8%) | 29 (56.9%, CI: 42.3% - 70.4%) | 0.147 | 29 (65.9%, CI: 50.0% - 79.1%) | 25 (55.6%CI: 40.1% - 70.0%) | 0.317 | **25 (39.1%, CI: 27.4% - 52.1%)** | **13 (21.7%, CI: 12.5% - 34.5%)** | **0.036** | **50 (78.1%, CI: 65.7% - 87.1%)** | **55 (98.2%, CI: 89.2% - 99.9%)** | **<0.001** | **158 (68.4%, CI: 61.9% - 74.3%)** | **123 (52.6%, CI: 46.0% - 59.1%)** | **<0.001** | **292 (65.6%, CI: 61.0% - 70.0%)** | **245 (54.9%, CI: 50.2% - 59.6%)** | **0.001** |
| Cephalexin | 0 (0.0%, CI: 0.0% - 53.7%) | 2 (4.1%, CI: 0.7% - 15.1%) | 1 | 0 (0.0%, CI: 0.0% - 53.7%) | 2 (4.5%, CI: 0.8% - 16.7%) | 1 | 0 (0.0%, CI: 0.0% - 40.2%) | 0 (0.0%, CI: 0.0% - 7.5%) | NA | 0 (0.0%, CI: 0.0% - 40.2%) | 3 (10.3%, CI: 2.7% - 28.5%) | 1 | 3 (4.8%, CI: 1.3% - 14.4%) | 1 (0.5%, CI: 0.0% - 2.9%) | 0.034 | 3 (3.4%, CI: 0.9% - 10.3%) | 8 (2.0%, CI: 0.9% - 4.0%) | 0.576 |
| ESBL-Producing | 4 (8.2%, CI: 2.6% - 20.5%) | 2 (3.9%, CI: 0.7% - 14.6%) | 0.432 | 1 (2.0%, CI: 0.1% - 11.8%) | 1 (2.2%, CI: 0.1% - 13.2%) | 1 | 0 (0.0%, CI: 0.0% - 6.6%) | 0 (0.0%, CI: 0.0% - 7.5%) | NA | 25 (32.9%, CI: 22.8% - 44.7%) | 27 (48.2%, CI: 34.8% - 61.8%) | 0.075 | **35 (12.5%, CI: 9.0% - 17.1%)** | **14 (6.0%, CI: 3.4% - 10.0%)** | **0.011** | 65 (12.4%, CI: 9.8% - 15.6%) | 44 (9.9%, CI: 7.3% - 13.1%) | 0.212 |
| Multidrug Resistance | 7 (16.7%, CI: 7.5% - 32.0%) | 9 (17.6%, CI: 8.9% - 31.4%) | 0.901 | 5 (11.4%, CI: 4.3% - 25.4%) | 1 (2.2%, CI: 0.1% - 13.2%) | 0.11 | 1 (1.6%, CI: 0.1% - 9.5%) | 1 (1.7%, CI: 0.1% - 10.1%) | 1 | 17 (26.6%, CI: 16.7% - 39.3%) | 21 (37.5%, CI: 25.2% - 51.5%) | 0.199 | 37 (16.0%, CI: 11.7% - 21.5%) | 32 (13.7%, CI: 9.7% - 18.9%) | 0.478 | 67 (15.1%, CI: 11.9% - 18.8%) | 64 (14.3%, CI: 11.3% - 18.0%) | 0.766 |
| Nitrofurantoin | 0 (0.0%, CI: 0.0% - 8.3%) | 0 (0.0%, CI: 0.0% - 8.7%) | 0.77 | 0 (0.0%, CI: 0.0% - 8.6%) | 0 (0.0%, CI: 0.0% - 9.8%) | 0.477 | 0 (0.0%, CI: 0.0% - 6.6%) | 0 (0.0%, CI: 0.0% - 7.5%) | 0.428 | 2 (2.6%, CI: 0.5% - 10.0%) | 2 (3.6%, CI: 0.6% - 13.4%) | 1 | 4 (1.4%, CI: 0.5% - 3.8%) | 3 (1.3%, 0.3% - 4.0%) | 1 | 6 (1.1%, CI: 0.5% - 2.5%) | 5 (1.1%, CI: 0.4% - 2.8%) | 0.998 |
| Trimethoprim-Sulfamethoxazole | 31 (57.4%, CI: 43.3% - 70.5%) | 20 (39.2%, CI: 26.2% - 53.9%) | 0.062 | 13 (24.1%, CI: 13.9% - 37.9%) | 5 (11.1%, CI: 4.2% - 24.8%) | 0.096 | 13 (18.3%, CI: 10.5% - 29.6%) | 8 (13.3%, CI: 6.3% - 25.1%) | 0.439 | 42 (53.2%, CI: 41.7% - 64.4%) | 29 (51.8%, CI: 38.2% - 65.2%) | 0.874 | **113 (38.6%, CI: 33.0% - 44.4%)** | **68 (29.1%, CI: 23.4% - 35.4%)** | **0.022** | **212 (38.5%, CI:** **34.4% - 42.7%)** | **130 (29.1%, CIL 25.0% - 33.6%)** | **0.002** |

Note: Counts of resistant isolates and percentages with confidence intervals (parenthesis). Confidence intervals are 95% confidence intervals of a proportion. For fluoroquinolones, isolates are considered resistant if they are resistant to at least one antibiotic of that class. P values are the results of pairwise chi-squared for resistance between 2019 and 2022 for that sequence type.
