# Supplementary material for: Trends in Uropathogenic Escherichia coli Genotype and Antimicrobial Resistance From 2019 to 2022 in a San Francisco Public Hospital Network
Source: Open Forum Infect Dis. 2025 Sep 17;12(9):ofaf579. doi: 10.1093/ofid/ofaf579 (PMC12464484; doi:10.1093/ofid/ofaf579)
Supplement: ofaf579_Supplementary_Data [file ofaf579_supplementary_data.zip › Supplemental_Table_5.docx]

Supplemental Table 5: Multivariable Logistic Regression Results for Antimicrobial Resistance

Note: table shows Odds Ratios and confidence intervals (parentheses) for each comorbidity/patient characteristic included in multivariable analyses for antimicrobial resistances. The dependent variable is the listed antimicrobial or resistance category. Results are displayed for the multivariable logistic regressions conducted among either all sequence types, or just within ST131.
